# Supplementary material for: Ultrastructural and Photosynthetic Responses of Pod Walls in Alfalfa to Drought Stress
Source: Int J Mol Sci. 2020 Jun 23;21(12):4457. doi: 10.3390/ijms21124457 (PMC7352927; doi:10.3390/ijms21124457)
Supplement: Supplementary file 1 [file ijms-21-04457-s001.zip › Supplementary file/Supplementary file 2.docx]

The different expression of proteins involved in the pathway related to photosynthesis under the pairwise comparison among development stages

|  |  |  | **G15 vs G10** | | **G20 vs G10** | | **G20 vs G15** | | **Z15 vs Z10** | | **Z20 vs Z10** | | **Z20 vs Z15** | |
| --- | --- | --- | --- | --- | --- | --- | --- | --- | --- | --- | --- | --- | --- | --- |
| **KEGG** | **Accession** | **Protein** | **Fold** | **P value** | **Fold** | **P value** | **Fold** | **P value** | **Fold** | **P value** | **Fold** | **P value** | **Fold** | **P value** |
| Photosynthesis –  antenna proteins | A0A072U9H4 | Chlorophyll a-b binding protein | 0.16 | 0.01 | NA | NA | NA | NA | 0.78 | 0.44 | 0.48 | NA | 0.61 | NA |
|  | I3SIG9 | Chlorophyll a-b binding protein | 0.3 | 0.02 | 0.4 | 0.04 | 1.35 | 0.05 | 0.42 | 0.01 | 0.04 | NA | 0.1 | NA |
|  | G7INT9 | Chlorophyll a-b binding protein | 3.88 | 0 | 1.11 | 0.61 | 0.29 | 0 | 1.19 | 0.59 | 0.24 | 0 | 0.2 | 0.04 |
|  | B7FIZ5 | Chlorophyll a-b binding protein | 0.96 | 0.65 | 0.22 | 0 | 0.23 | 0 | 0.22 | 0 | 0.44 | 0 | 1.98 | 0.03 |
|  | G7JB75 | Chlorophyll a-b binding protein | 0.61 | 0.12 | NA | NA | NA | NA | 0.12 | 0.01 | 0.71 | NA | 5.92 | NA |
|  | I3SZG9 | Chlorophyll a-b binding protein | 0.58 | 0.06 | NA | NA | NA | NA | 0.53 | 0 | 0.3 | 0 | 0.57 | 0.08 |
|  |  |  |  |  |  |  |  |  |  |  |  |  |  |  |
| Carbon fixation in photosynthetic  organisms | A0A072TYY4 | Ribulose-phosphate 3-epimerase | 2.19 | 0.04 | 4 | 0.01 | 1.82 | 0.03 | 2.81 | 0.14 | NA | NA | NA | NA |
|  | A0A072TPE4 | 26S proteasome non-ATPase regulatory subunit 6 | 0.71 | 0.01 | 0.48 | 0.01 | 0.68 | 0.02 | 0.61 | 0.3 | 0.83 | 0.26 | 1.36 | 0.56 |
|  | A0A072VCX2 | Aspartate aminotransferase | 1.42 | 0.01 | 0.53 | 0 | 0.37 | 0 | 0.56 | 0.03 | 1 | 0.99 | 1.8 | 0.06 |
|  | G8A0S6 | Cytosolic fructose-1 6-bisphosphatase | 2.71 | 0 | 1.46 | 0.17 | 0.54 | 0 | 1.15 | 0.08 | 1.47 | NA | 1.28 | NA |
|  | I3S3S0 | Cytosolic triosephosphate isomerase | 1.08 | 0.38 | 1.02 | 0.77 | 0.95 | 0.56 | 0.89 | 0.18 | 0.46 | 0 | 0.52 | 0.02 |
|  | G7JV43 | Fructose-1,6-bisphosphatase | 3.12 | 0 | 1.86 | NA | 0.6 | NA | 0.93 | NA | 1.75 | 0.54 | 1.88 | NA |
|  | A0A072U1Q8 | Fructose-1,7-bisphosphatase | 1.83 | 0.07 | 2.76 | 0.01 | 1.51 | 0.06 | 2.75 | 0.01 | 1.35 | 0.51 | 0.49 | 0.09 |
|  | A0A072VVG3 | Fructose-bisphosphate aldolase | 16.09 | 0.05 | 38.12 | 0 | 2.37 | 0.01 | 3.97 | 0.19 | NA | NA | NA | NA |
|  | I3SU63 | Fructose-bisphosphate aldolase | 3.02 | 0 | 3.41 | 0 | 1.13 | 0.18 | 2.61 | 0.01 | 1.76 | 0.28 | 0.67 | 0.29 |
|  | G7K4T4 | Fructose-bisphosphate aldolase | 2.68 | 0.02 | 6.81 | 0.06 | 2.54 | 0.13 | 4.93 | 0.09 | NA | NA | NA | NA |
|  | G7JYY7 | Glutamate-glyoxylate aminotransferase | 1.67 | 0 | 0.64 | 0.1 | 0.38 | 0 | 1.11 | 0.76 | 1.4 | 0.27 | 1.26 | 0.53 |
|  | G7J2H2 | Glyceraldehyde-3-phosphate dehydrogenase | 0.52 | 0 | 0.68 | 0.02 | 1.3 | 0.13 | 0.33 | 0 | 0.63 | 0.01 | 1.91 | 0.03 |
|  | G7JTZ0 | Glyoxysomal malate dehydrogenase | 0.87 | 0.63 | 4.58 | 0.05 | 5.26 | 0.05 | 1.14 | 0.43 | 3.78 | NA | 3.32 | NA |
|  | B7FJQ4 | Malate dehydrogenase | 1.08 | 0.86 | 3.33 | 0 | 3.1 | 0.02 | 1.49 | 0.32 | NA | NA | NA | NA |
|  | A0A072VMC4 | Malate dehydrogenase | 1.87 | 0.02 | 4.01 | 0 | 2.15 | 0 | 1.39 | 0.09 | 0.43 | NA | 0.31 | NA |
|  | A0A072TQ67 | Malate dehydrogenase | 1.91 | 0 | 3.09 | 0 | 1.62 | 0 | 1.46 | 0.32 | 1.7 | 0.08 | 1.16 | 0.66 |
|  | A0A072VD34 | Malic enzyme | NA | NA | NA | NA | NA | NA | 2.57 | 0.02 | NA | NA | NA | NA |
|  | G7L7H0 | Malic enzyme | 4.46 | 0 | 6.65 | 0 | 1.49 | 0.11 | 2.36 | 0.05 | 1.75 | 0.23 | 0.74 | 0.44 |
|  | G7IU25 | Phosphoenolpyruvate carboxylase | 2.15 | 0.08 | 2.01 | 0.12 | 0.94 | 0.86 | NA | NA | 6.07 | 0.03 | NA | NA |
|  | G7IT86 | Phosphoglycerate kinase | 0.66 | 0.09 | 0.92 | 0.6 | 1.41 | 0.19 | 0.45 | 0.04 | 0.3 | 0.03 | 0.66 | 0.35 |
|  | G7IT85 | Phosphoglycerate kinase | 0.33 | 0 | 0.54 | 0.01 | 1.62 | 0.07 | 0.22 | 0 | 0.18 | 0 | 0.79 | 0.57 |
|  | G7L1U4 | Ribose-5-phosphate isomerase A | 1.2 | 0.02 | 0.86 | 0.16 | 0.72 | 0.01 | 0.87 | 0.55 | 1.33 | 0.28 | 1.53 | 0.23 |
|  | G7JAP0 | Sedoheptulose-1,7-bisphosphatase | 1.5 | 0.03 | 2.12 | 0.02 | 1.41 | 0.08 | 0.99 | 0.92 | 1.25 | 0.7 | 1.26 | 0.78 |
|  |  |  |  |  |  |  |  |  |  |  |  |  |  |  |
| Photosynthesis | G7JFY7 | Cytochrome b559 subunit alpha | 0.05 | 0.05 | 0.89 | 0.77 | 16.42 | 0 | 1.32 | 0.42 | 0.2 | 0.07 | 0.15 | 0.05 |
|  | B7FGU7 | Cytochrome b6-f complex iron-sulfur subunit | 0.55 | 0.05 | 1.15 | 0.43 | 2.09 | 0 | 1.58 | 0.13 | 0.3 | 0.02 | 0.19 | 0.01 |
|  | A0A072V4G2 | F0F1 ATP synthase subunit gamma | 1.51 | 0.09 | 2.25 | 0 | 1.49 | 0.03 | 2.46 | 0.01 | 2.68 | 0.09 | 1.09 | 0.8 |
|  | G7J4F9 | Light-harvesting complex I chlorophyll a-b binding protein | 0.73 | 0.08 | 0.11 | 0 | 0.15 | 0.04 | 0.4 | 0.01 | 1.19 | 0.79 | 2.98 | 0.31 |
|  | A0A072TYH7 | Oxygen-evolving complex/thylakoid lumenal 25.6 kDa protein | 1.25 | 0.39 | 3.23 | 0.05 | 2.58 | 0.06 | 2.98 | 0 | 1.11 | NA | 0.37 | NA |
|  | B7FJ16 | Oxygen-evolving enhancer protein 2-1 | 0.43 | 0.07 | 0.57 | NA | 1.32 | NA | 0.51 | 0 | 0.12 | NA | 0.23 | NA |
|  | I3SSE5 | Oxygen-evolving enhancer protein | 0.32 | 0.03 | 0.46 | 0.07 | 1.43 | 0.19 | 0.36 | 0.02 | 0.17 | 0 | 0.48 | 0.1 |
|  | G7ZVI4 | Oxygen-evolving enhancer protein | 0.14 | 0.13 | 0.08 | NA | 0.58 | NA | 0.26 | 0 | NA | NA | NA | NA |
|  | G7K9H5 | Photosystem II oxygen-evolving enhancer protein | 2.76 | 0.04 | NA | NA | NA | NA | 1.22 | 0.65 | NA | NA | NA | NA |
|  | G7J0Z5 | Photosystem I P700 chlorophyll a apoprotein a2 | 0.6 | 0.01 | 0.3 | 0 | 0.51 | 0.01 | 0.92 | 0.25 | 0.94 | 0.44 | 1.02 | 0.66 |
|  | G7K2D0 | Photosystem I reaction center subunit II | 0.26 | 0.21 | 1.06 | NA | 4.04 | NA | 1.27 | 0.09 | NA | NA | NA | NA |
|  | G7JAX6 | Photosystem I reaction center subunit N | NA | NA | NA | NA | NA | NA | 4.16 | NA | NA | NA | NA | NA |
|  | A0A072TKG2 | Photosystem II D2 protein | 1.13 | 0.6 | 0.34 | 0.01 | 0.3 | 0.04 | 0.71 | 0 | 0.06 | 0 | 0.09 | 0 |

Z10, Z15 and Z20 respectively means pod wall on DAP10, DAP15 and DAP20 under well-watered treatments; G10, G15 and G20 respectively means pod wall on on DAP10, DAP15 and DAP20 under water-stressed treatments. NA, not applicable.
