# Supplementary material for: Ultrastructural and Photosynthetic Responses of Pod Walls in Alfalfa to Drought Stress
Source: Int J Mol Sci. 2020 Jun 23;21(12):4457. doi: 10.3390/ijms21124457 (PMC7352927; doi:10.3390/ijms21124457)
Supplement: Supplementary file 1 [file ijms-21-04457-s001.zip › Supplementary file/Supplementary file 3.docx]

The different expression of proteins involved in the pathway related to photosynthesis under the comparison between water-stressed and well-watered treatments

| **Patyway** | **Accession** | **Protein** | **G10 vs Z10** | | **G15 vs Z15** | | **G20 vs Z20** | |
| --- | --- | --- | --- | --- | --- | --- | --- | --- |
|  |  |  | **Fold** | **P value** | **Fold** | **P value** | **Fold** | **P value** |
| Photosynthesis –  antenna proteins | A0A072U9H4 | Chlorophyll a-b binding protein | 1.02 | 0.94 | 0.21 | 0.09 | NA | NA |
|  | I3SIG9 | Chlorophyll a-b binding protein | 0.45 | 0.01 | 0.32 | 0.05 | 4.48 | NA |
|  | G7INT9 | Chlorophyll a-b binding protein | 0.35 | 0 | 1.13 | 0.64 | 1.58 | 0.33 |
|  | B7FIZ5 | Chlorophyll a-b binding protein | 0.52 | 0 | 2.26 | 0.01 | 0.26 | 0.01 |
|  | G7JB75 | Chlorophyll a-b binding protein | 0.61 | 0.11 | 3.07 | 0.01 | NA | NA |
|  | I3SZG9 | Chlorophyll a-b binding protein | 0.45 | 0 | 0.5 | 0.01 | NA | NA |
|  |  |  |  |  |  |  |  |  |
| Carbon fixation in photosynthetic organisms | A0A072TYY4 | Ribulose-phosphate 3-epimerase | 0.86 | 0.69 | 0.67 | 0.41 | NA | NA |
|  | A0A072TPE4 | 26S proteasome non-ATPase regulatory subunit 6 | 1.13 | 0.16 | 1.3 | 0.59 | 0.65 | 0.11 |
|  | A0A072VCX2 | Aspartate aminotransferase | 1.1 | 0 | 2.82 | 0 | 0.59 | 0.02 |
|  | G8A0S6 | Cytosolic fructose-1 6-bisphosphatase | 0.36 | 0 | 0.84 | 0.05 | 0.35 | NA |
|  | I3S3S0 | Cytosolic triosephosphate isomerase | 0.98 | 0.75 | 1.2 | 0.13 | 2.17 | 0.01 |
|  | G7JV43 | Fructose-1,6-bisphosphatase | 0.38 | 0 | 1.28 | NA | 0.41 | NA |
|  | A0A072U1Q8 | Fructose-1,7-bisphosphatase | 0.66 | 0.1 | 0.44 | 0.02 | 1.36 | 0.39 |
|  | A0A072VVG3 | Fructose-bisphosphate aldolase | 0.13 | 0.04 | 0.53 | 0.27 | NA | NA |
|  | I3SU63 | Fructose-bisphosphate aldolase | 0.65 | 0.09 | 0.75 | 0.15 | 1.25 | 0.5 |
|  | G7K4T4 | Fructose-bisphosphate aldolase | 0.48 | 0.09 | 0.26 | 0.1 | NA | NA |
|  | G7JYY7 | Glutamate-glyoxylate aminotransferase | 1.09 | 0.51 | 1.64 | 0.08 | 0.5 | 0.11 |
|  | G7J2H2 | Glyceraldehyde-3-phosphate dehydrogenase | 1.05 | 0.49 | 1.67 | 0.02 | 1.14 | 0.49 |
|  | G7JTZ0 | Glyoxysomal malate dehydrogenase | 0.65 | 0.12 | 0.5 | 0.02 | 0.79 | NA |
|  | B7FJQ4 | Malate dehydrogenase | 1.05 | 0.89 | 0.76 | 0.56 | NA | NA |
|  | A0A072VMC4 | Malate dehydrogenase | 0.56 | 0.02 | 0.76 | 0.14 | 5.23 | NA |
|  | A0A072TQ67 | Malate dehydrogenase | 1 | 0.98 | 1.3 | 0.35 | 1.81 | 0.02 |
|  | A0A072VD34 | Malic enzyme | 3.04 | 0.01 | NA | NA | NA | NA |
|  | G7L7H0 | Malic enzyme | 0.37 | 0 | 0.7 | 0.24 | 1.41 | 0.32 |
|  | G7IU25 | Phosphoenolpyruvate carboxylase | 1.82 | 0.07 | NA | NA | 0.6 | 0.34 |
|  | G7IT86 | Phosphoglycerate kinase | 1.17 | 0.33 | 1.72 | 0.21 | 3.66 | 0.02 |
|  | G7IT85 | Phosphoglycerate kinase | 1 | 0.97 | 1.47 | 0.25 | 3.01 | 0.01 |
|  | G7L1U4 | Ribose-5-phosphate isomerase A | 0.63 | 0 | 0.87 | 0.61 | 0.41 | 0.04 |
|  | G7JAP0 | Sedoheptulose-1,7-bisphosphatase | 0.68 | 0.01 | 1.04 | 0.82 | 1.16 | 0.83 |
|  |  |  |  |  |  |  |  |  |
| Photosynthesis | G7JFY7 | Cytochrome b559 subunit alpha | 0.6 | 0.25 | 0.02 | 0.01 | 2.69 | 0.05 |
|  | B7FGU7 | Cytochrome b6-f complex iron-sulfur subunit | 1.07 | 0.77 | 0.37 | 0.02 | 4.13 | 0 |
|  | A0A072V4G2 | F0F1 ATP synthase subunit gamma | 0.75 | 0.05 | 0.46 | 0.02 | 0.63 | 0.26 |
|  | G7J4F9 | Light-harvesting complex I chlorophyll a-b binding protein | 1.09 | 0.3 | 2.01 | 0.11 | 0.1 | 0.3 |
|  | A0A072TYH7 | Oxygen-evolving complex/thylakoid lumenal 25.6 kDa protein | 0.97 | 0.86 | 0.41 | 0 | 2.82 | NA |
|  | B7FJ16 | 2-1 Oxygen-evolving enhancer protein 2-1 | 0.47 | 0 | 0.4 | 0.06 | 2.32 | NA |
|  | I3SSE5 | Oxygen-evolving enhancer protein | 0.72 | 0.25 | 0.64 | 0.21 | 1.93 | 0.08 |
|  | G7ZVI4 | Oxygen-evolving enhancer protein | 0.47 | 0.03 | 0.25 | 0.22 | NA | NA |
|  | G7K9H5 | Photosystem II oxygen-evolving enhancer protein | 0.15 | 0 | 0.34 | 0.26 | NA | NA |
|  | G7J0Z5 | Photosystem I P700 chlorophyll a apoprotein a2 | 0.77 | 0.03 | 0.5 | 0 | 0.25 | 0 |
|  | G7K2D0 | Photosystem I reaction center subunit II | 0.46 | 0.04 | 0.1 | 0.01 | NA | NA |
|  | G7JAX6 | Photosystem I reaction center subunit N | 0.43 | 0.01 | NA | NA | NA | NA |
|  | A0A072TKG2 | Photosystem II D2 protein | 0.55 | 0 | 0.87 | 0.51 | 3.04 | 0.18 |

Z10, Z15 and Z20 respectively means pod wall on DAP10, DAP15 and DAP20 under well-watered treatments; G10, G15 and G20 respectively means pod wall on on DAP10, DAP15 and DAP20 under water-stressed treatments. NA, not applicable.
